# Retinal Ganglion Cell Transplantation: Approaches for Overcoming Challenges to Functional Integration

**DOI:** 10.3390/cells10061426

**Published:** 2021-06-08

**Authors:** Kevin Y. Zhang, Erika A. Aguzzi, Thomas V. Johnson

**Affiliations:** Glaucoma Center for Excellence, Wilmer Eye Institute, Johns Hopkins University School of Medicine, 600 North Wolfe Street, Maumenee B-110, Baltimore, MD 21287, USA; yzhan427@jhmi.edu (K.Y.Z.); eaguzzi1@jhmi.edu (E.A.A.)

**Keywords:** retinal ganglion cell, optic nerve, neuron, transplantation, regeneration, engraftment, functional integration, stem cells, cell replacement, regenerative medicine

## Abstract

As part of the central nervous system, mammalian retinal ganglion cells (RGCs) lack significant regenerative capacity. Glaucoma causes progressive and irreversible vision loss by damaging RGCs and their axons, which compose the optic nerve. To functionally restore vision, lost RGCs must be replaced. Despite tremendous advancements in experimental models of optic neuropathy that have elucidated pathways to induce *endogenous* RGC neuroprotection and axon regeneration, obstacles to achieving functional visual recovery through *exogenous* RGC transplantation remain. Key challenges include poor graft survival, low donor neuron localization to the host retina, and inadequate dendritogenesis and synaptogenesis with afferent amacrine and bipolar cells. In this review, we summarize the current state of experimental RGC transplantation, and we propose a set of standard approaches to quantifying and reporting experimental outcomes in order to guide a collective effort to advance the field toward functional RGC replacement and optic nerve regeneration.

## 1. Introduction

**Figure 1:**
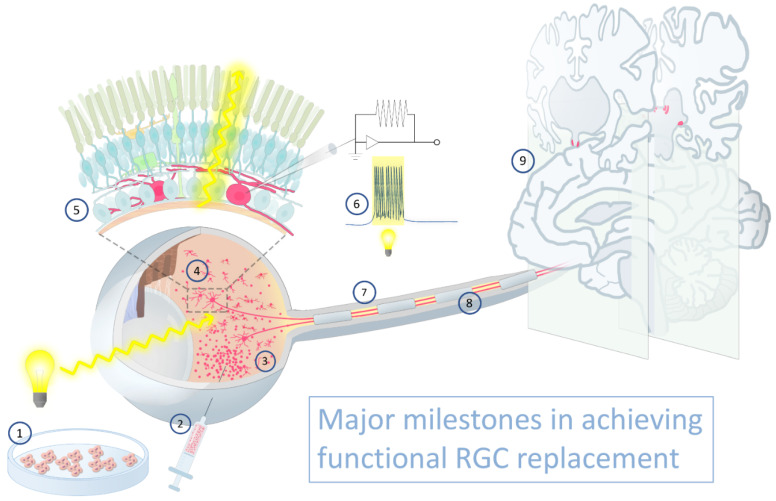
Major milestones in achieving functional RGC replacement: (**1**) Develop a reliable source of transplantable RGCs. (**2**) Deliver donor cells (depicted in red) safely. (**3**) Promote long-term donor RGC survival in the recipient eye. (**4**) Establish retinal localization and neuritogenesis. (**5**) Form synaptic connectivity with host retinal interneurons in the IPL. (**6**) Conduct light-evoked, photoreceptor-transduced, signals within the visual pathway. (**7**) Achieve axon growth toward the optic nerve head and into the optic nerve. (**8**) Ensure myelination of new axons. (**9**) Reinnervate retinorecipient nuclei, including suprachiasmatic nucleus, lateral geniculate nucleus, olivary pretectal nucleus, and superior colliculus.

The retina is embryologically derived from central nervous system (CNS) neuroectodermal progenitors. The mammalian retina, therefore, like the rest of the mammalian CNS, lacks inherent regenerative capacity [1]. Retinal ganglion cells (RGCs) are the projection neurons of the retina, with somas localized to the RGC layer (RGCL); dendrites that synapse with bipolar and amacrine cells within the inner plexiform layer (IPL); and axons that traverse the retinal nerve fiber layer (RNFL), the optic nerve, and the optic tracts before eventually synapsing within an array of central targets (Figure 1). Optic neuropathies, including glaucoma, are characterized by progressive RGC death, which results in irreversible vision loss [2]. Pharmacologic and surgical treatments for glaucoma slow disease worsening by reducing intraocular pressure (IOP) [3]; however, their efficacy is limited by side effects, suboptimal patient adherence to therapy, surgical complications, and the potential for disease progression despite significant IOP reduction [4,5,6,7]. Similarly, limited interventions are capable of slowing or halting progression of optic neuropathies occurring secondary to ischemia, inflammation, toxic or metabolic insults, and inherited gene defects. No existing therapy can reverse blindness following RGC loss. Even emerging neuroprotective strategies designed to modulate intracellular signaling pathways to improve cell survival and preserve remaining RGCs following initial neuronal insult [8,9] will not restore vision already lost due to RGC death. To achieve functional restoration of vision, RGC replacement is required [10].

Retinal diseases have been pioneering targets for therapeutic cell transplantation in medicine [11,12], with retinal pigment epithelial cell and photoreceptor transplantation at various stages of clinical development [13,14,15]. However, RGC replacement faces numerous challenges unique to the inherent complexity of this neuronal cell type. Subretinal injection normally performed for experimental photoreceptor transplantation may be less applicable to RGC replacement because the transplanted RGCs would need to migrate through the entire retina to localize properly into the host RGCL and extend axons within the RNFL. Intravitreal transplantation might provide more direct access to the inner retina, but introduces new obstacles including dispersion of cells into a much larger three-dimensional space, without sequestration adjacent to retinal tissue, and behind physical barriers not present in the subretinal space. The internal limiting membrane (ILM) at the vitreoretinal junction impedes transplanted RGC migration into the host retina [16] and should be mitigated in a safe manner. Once localized to the host RGCL, donor RGCs must establish proper topographical spacing, while targeting dendrites to the relevant IPL sublamina to form synaptic connections with amacrine and bipolar cells. Finally, donor RGCs must extend axons through the optic nerve and reinnervate CNS targets in a retinotopic manner. To further complicate the task, dozens of unique RGC subtypes exist in rodents and primates, each with specific pre- and post-synaptic patterns of connectivity [17]. Moreover, functional transmission of action potentials within the visual pathway depends on axonal myelination within the optic nerve. Thus, functional replacement with donor RGCs will require a concerted effort to address multiple complex challenges (Figure 1).

Molecular targets relevant to achieving critical milestones in functional RGC replacement, particularly the achievement of RGC survival and axon regeneration, have been elucidated by studying *endogenous* RGCs following experimental injury [18]. For example, intrinsic mTOR and CNTF/JAK/STAT signaling promote RGC survival and axon regeneration [19,20]. Insulin signaling [21], overexpression of Lin28 [22], induction of thrombospondin-1 [23], deletion of PTEN and SOCS3 [24,25,26], and inhibition of KLF [27] in endogenous RGCs all augment axon regeneration in various traumatic optic neuropathy models. Additionally, over 40 negative intrinsic regulators of RGC regeneration have now been identified [28]. RGC-extrinsic signaling molecules, including oncomodulin, also promote axon regeneration in disease models [23,29]. Moreover, long-distance regenerated RGC axons can be remyelinated [30], establish synaptic connectivity with retinorecipient nuclei, and restore visually guided behaviors in mice [31].

By comparison, in vivo transplantation of exogenous primary RGCs or stem cell derived RGCs remains a field in its infancy. Nonetheless, pioneering transplantation studies have recorded light-evoked electrophysiological responses from donor RGCs [32] and documented improvements in visually guided behaviors in recipient animals [33], providing a proof of principle for the therapeutic potential of RGC transplantation. In contrast to the extensive body of work characterizing RGC axon regeneration and efferent reconnectivity following injury, data guiding methods to enhance donor cell survival, attain retinal localization, or achieve afferent synaptogenesis of replacement RGCs are limited and will be the emphasis of this review. To aid in propelling this field towards robust and reproducible evaluation and reporting of these outcomes, we offer suggested approaches to validating transplanted RGC survival, migration and topographical spacing, dendrite extension and localization, and synaptogenesis within the IPL.

## 2. RGC Sources for Transplantation

RGC transplantation strategies require generating lineage committed progenitors or terminally differentiated neurons in sufficiently large quantities. The source of donor cells for transplantation (Table 1) into human patients should be human derived, scalable, and capable of bona fide RGC differentiation. As such, most efforts to create transplantable donor cells have concentrated on human embryonic stem cell (hESC) and induced pluripotent stem cell (iPSC) derived RGCs. Differentiation methodologies to generate RGCs have been described by numerous protocols and comprehensive reviews [34,35,36,37]. Here, we will only briefly highlight the major developmental pathways and transcriptional events relevant to RGC lineage specification.

ESCs are derived from the inner cell mass of embryonic blastocysts [38], and they were the first cell type used to generate retinal progenitor cells (RPCs) [39]. Differentiated RPCs recapitulate the major transcriptional regulators of RGC lineage specification by expressing Pax6, RX, and Math5 [40]. Further lineage commitment into RGCs is achieved by mirroring developmental pathways, including those of IGF-1, BMP, Wnt, and Notch [41,42,43]. For example, DAPT, an inhibitor of Notch signaling, downregulates this pathway to release inhibition of RGC differentiation [44], and is sufficient to produce RGCs that express Tuj1, BRN3a, Islet-1, Thy1, and gamma-synuclein [45,46,47]. Concurrent activation of IGF and inhibition of Wnt signaling by IGF1 and DKK1/Noggin, respectively, further specifies cells with RGC-like gene expression patterns [48]. CRISPR-Cas9 engineered stem cell lines expressing *BRN3b*-dependent tdTomato and murine *Thy1.2* have facilitated purification of RGCs and longitudinal cell tracking in transplantation studies [36].

Adult human somatic cells, after appropriate induction, can dedifferentiate and resemble ESCs in morphology, and have the capacity for multilineage differentiation and self-renewal [49]. When autologous in origin, iPSCs have a low risk of host rejection, and their use is unencumbered by ethical concerns surrounding human embryo destruction. Numerous protocols exist to direct iPSCs to RPCs and RGC-like cells [34,50,51,52]. Undifferentiated iPSCs show retinal neuron potentiality when stimulated by DKK1, Noggin, and DAPT, the same factors used in hESC differentiation [53]. Once differentiated, iPSC-RGCs express canonical RGC markers such as Brn3a, Brn3b, Islet1, and Thy1 [52,54].

A limitation to cellular differentiation in two dimensional culture is a lack of multi-cellular organization, which is overcome by three dimensional cultures of self-organizing organoids [55]. These “organs in a dish” provide a deeper insight into tissue morphogenesis and maturation by recapitulating the complex spatiotemporal interplay between different cell types and their microenvironment during development. Mouse retinal organoids were first generated by culturing ESCs in a 3D suspension of embryoid-body aggregates that self-organized into layered structures resembling the embryonic optic cup [56]. Differentiated cells in the 3D structure express RX, the retinal homeobox gene that is critical in optic vesicle invagination and retinal layering [57]. Continued culture for one week leads to dynamic shape change and expression of neuroretinal markers. Long-term culturing beyond 7 to 22 weeks leads to spontaneous differentiation of RPCs into all the major neural retinal cell types in their proper layers [58]. Remarkably, the organoid-derived photoreceptors exhibit sporadic responses to light, indicating their functional maturation. Promising reports have identified protocols for generating and isolating human organoid-derived RGCs [59], providing new possibilities in developing donor sources for cell replacement therapies. 

Taken together, characterization of robust RGC differentiation from multiple cell lineages and culture conditions have paved the way for stem cell-based replacement strategies as a promising approach to optic nerve regeneration. The tools now exist to study, in earnest, RGC transplantation in vivo. 

In order to maximize generalization of protocols and experimentation worldwide, standard descriptions of donor cell characteristics are critical when reporting RGC transplantation results. Such studies should explicitly describe the parental stem cell line or source, differentiation culture conditions, the stage of RGC or progenitor at harvest, RGC purification technique and yield, and the expression of a standard set of canonical RGC markers. Ideally, critical results should be replicated with multiple independent lines of donor RGCs. Critically, we do not know the developmental state for donor cells—whether fully mature RGCs, developing RGCs, or lineage-committed progenitors—that will yield the best engraftment results. Whether stem cells or organoids are used as a source of donor RGCs, the isolation process should maximize purity in order to limit aberrant tissue overgrowth or teratoma formation from undifferentiated cell populations. Furthermore, RGCs survive poorly in organoids during prolonged culture [60], presumably due to the absence of retrograde trophic support from the CNS, which may limit their potential to generate large numbers of RGCs without additional modifications. Guided RGC subtype specification, which has been described in both two-dimensional and organoid cultures [61,62], would allow for a more refined approach to future stem cell-based treatments.

## 3. Transplanted RGC Survival

A critical prerequisite to studying interactions between donor RGCs and the host retina is robust survival of transplanted neurons. Unfortunately, this has been a formidable obstacle in most studies to date (see below). One of the difficulties in comparing survival rates across multiple methodologies stems from unstandardized methods of reporting survival, either as an absolute number, a percentage of transplanted cells, or by providing the density of surviving cells. The transplantation route plays a critical role in discerning donor cell survival. Administration to the basal (vitreous) side of the retina via intravitreal injection provides the most direct access to the inner retina, without the need for potentially disruptive transparenchymal migration [63]. Moreover, such methodology may minimize damage to the blood-retinal barrier (BBB), especially in humans where the posterior segment can be accessed via the pars plana. However, intravitreal injection disperses cells into a large cavity. As homing and integration of injected cells to retinal targets cannot be accurately controlled at present, grafted cells have the potential to distribute broadly throughout the posterior segment of the eye, and often do, including on the posterior lens capsule and the ciliary body [64]. Therefore, donor cell survival within the eye should examine all ocular tissues, or the metric should be qualified as a composite outcome of survival and retinal homing. The kinetics and patterns of injected cell migration and survival are an understudied aspect of intraocular transplantation.

### 3.1. Assessing Donor RGC Survival

In nearly all cases, where reported, intraocular transplantation of RGCs has yielded low rates of neuronal survival, far below what would be required to restore visual function in advanced optic neuropathy (Table 2). Development of innovative approaches for improving transplanted RGC survival in the eye remains a fundamental challenge that must be met before optimization of functional neural integration can begin in earnest. The average number of RGCs per eye in healthy adult mammals varies by species and is correlated to eye size. Mice and rats have approximately 50,000 and 80,000 cells per eye [65,66], respectively, whereas humans have between 600,000 to 1.2 million RGCs per eye [67]. Studies of human and primate eyes that have correlated postmortem structure to visual function during life suggest, however, that meaningful improvements in vision could be attained by replacing just a fraction of the normal RGC number. In human patients, 25% to 35% RGC loss is associated with mild local visual abnormalities identified by automated perimetry, which suggests that near-normal vision can be supported by only 65% of the normal RGC number [67]. Estimates using noninvasive optical coherence imaging of the retinal nerve fiber layer similarly suggest that up to 57% of RGCs can be lost before visual field defects are detectable [68]. Similarly, a relative afferent pupillary defect does not manifest until at least 25% of RGCs are lost [69]. Whereas the relationship between visual function and RGC number is unlikely to be linear, and these figures may not translate directly between species, it is not unreasonable as a first estimate to expect that a meaningful improvement in vision could be attained by replacement of 10–20% of RGCs. Such a level of replacement may not confer the ability to read fine print, but for a patient without light perception, even the ability to see shapes and movements could have dramatic ramifications for quality of life. In a mouse, this might equate to 5000–10,000 RGCs. To date, where quantifications have been reported, the survival of transplanted RGCs at even relatively short time points of 1–4 week are typically much less than 5000 RGCs (Table 2).

Purified postnatal mouse RGCs transplanted into rat recipients survive at a typical rate of about 1% [32], although the results show a high degree of variation among eyes. Co-transplantation of iPSCs with purified mouse RGCs by the same group marginally improves donor RGC survival in adult rat retinas, presumably by providing trophic support to the co-injected RGCs [76]. hESC-derived RGCs survive for at least one week in healthy rat eyes, with a published surviving donor cell density of 19–25 cells/mm^2^ [71]. However, reporting survival as cell density rather than as an absolute number of transplanted cells makes comparisons of survival efficiency challenging. If we estimate the numerical survival by multiplying the density by the average surface area of a rat retina of 80 mm^2^ [77] (which presumes 100% coverage of the retinal surface are by donor cells) then we calculate a maximum overall survival of 1520–2000 cells per eye, or approximately 4% of the 50,000 transplanted donor cells. While these studies are, to the best of our knowledge, the only ones to report quantitative donor RGC survival data post transplantation, they serve to set the benchmark for future studies.

**Figure 2:**
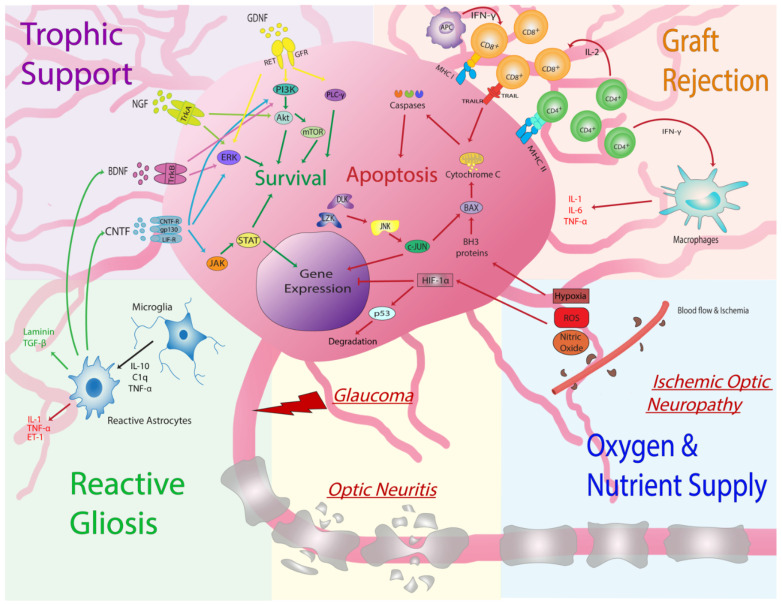
Cell intrinsic and environmental processes and signaling pathways regulating RGC survival following transplantation. Akt—protein kinase B; BAX—bcl-2-like protein 4; APC—antigen presenting cell; BDNF—brain derived neurotrophic factor; C—complement; CD—cluster of differentiation; CNTF—ciliary neurotrophic factor; DLK—dual leucine zipper kinase; ERK—extracellular signal-regulated kinase; ET—endothelin; GDNF—glial derived neurotrophic factor; GFR—GDNF family receptor; gp130—Glycoprotein 130 receptor; HIF—hypoxia inducible factor; IFN— interferon; IL—interleukin; JAK—Janus kinase; JNK—c-Jun-N-terminal kinase; LIF—leukemia inhibitor factor; LZK—leucine zipper kinase; MHC—major histocompatibility complex; NGF—nerve growth factor; p53—transformation-related protein 53; PI3K—phosphoinositide 3-kinase; PLC—phospholipase C; mTOR—mammalian target of rapamycin; RET—rearranged during transfection receptor; ROS—reactive oxygen species; STAT—signal transducer and activator of transcription proteins; TNF—tumor necrosis factor; Trk—tropomyosin receptor kinase.

In order to increase transplanted RGC survival from low single digit rates, accurately assessing incremental improvements at early stages of RGC transplantation is necessary. Several factors may influence RGC survival. In addition to cell intrinsic signaling pathways and environmental influences (Figure 2, discussed below), the number of cells transplanted may directly influence survival rates [32]. In mouse eyes, 2–5 × 10^4^ RGCs are typically transplanted per eye, but the optimal number should be determined empirically for individual sources of donor cells and specific recipient animals and model systems. As resources permit, multiple time points should be assessed to determine the survival kinetics of transplanted cells, as the rate of donor RGC death is likely nonlinear over time. As various donor cell transplantation methodologies are evaluated, we offer the following recommendations as a standardized approach to reporting donor cell survival:

- When numbers of surviving donor neurons are low, quantification is ideally performed on retinal flatmounts such that the entire retinal surface area is examined. Histological sections are prone to sampling errors unless graft neurons are evenly spaced. Tiled microscopy of the entire host retinal surface should be imaged at high resolution;
- If donor cells express a fluorescent marker that is cytoplasmic and contained within neurites, colocalization with a secondary nuclear marker is recommended in order to distinguish overlapping donor cells. If human cells are transplanted into nonhuman recipients, antibodies directed against human nuclear antigen may be suitable;
- If donor cells fail to disperse after transplantation, the resulting cell clump is best imaged as a confocal z-stack in order to parse overlapping cells.

### 3.2. Increasing Donor RGC Survival: Cell Intrinsic Signaling Pathways

Intrinsic molecular signaling pathways ultimately drive neuronal survival during development and following stress or injury. Broadly speaking, multiple pathways converge to activate or inhibit caspase-mediated apoptosis, and strategies that modulate these signaling pathways may improve the survival of transplanted RGCs. The mammalian target of rapamycin (mTOR) pathway is particularly relevant to RGC survival and regeneration following injury [24]. Downregulation of mTOR occurs following axotomy, and mTOR inhibitors upregulate just prior to RGC death [78]. Conversely, induced mTOR expression via transgenic manipulation is neuroprotective after optic nerve damage [79]. Differences in relative mTOR expression across different RGC subtypes may explain the enhanced resiliency and regenerative potential exhibited by the alpha-RGCs [80]. BDNF (see below), some essential amino acids, glucose, leptin and sphingosine-1-phosphate (S1P), and GβL protein are all mTOR modulating molecules that confer neuroprotection [21,81,82]. In addition, the PI3K/Akt/mTOR pathway is an important cell cycle regulator. PI3K/Akt enhances the activity of cell survival pathways and has anti-apoptotic effects, particularly under conditions of stress, such as withdrawal of trophic factors, oxidative stress, and ischemic shock [83]. Phosphatase and tensin homologue (PTEN) antagonize PI3K activity and is therefore an Akt and mTOR inhibitor. Inactivation of PTEN enhances Akt signaling, suppresses apoptosis, and promotes axonal regeneration in RGCs and other neurons [84]. 

Dual leucine zipper kinase (DLK) and leucine zipper kinase (LZK) are other key sensors in the RGC injury response [9]. Upon damage to RGCs, DLK and LZK cooperate to activate mitogen-activated protein kinase kinases (MKK)-4 and -7, which in turn activate Jun-N-terminal kinases 1-3 (JNK1-3), and important downstream pro-apoptotic mediators including c-Jun [85]. The *Bax* subfamily of Bcl-2-related proteins is essential for JNK-dependent apoptosis [86], and its inhibition significantly reduces RGC loss following injury [87]. Initiation of transcriptional stress responses occurs promptly in RGCs and includes interactions of DLK with JNK-interacting protein 3 (JIP3) and MKK7, leading to phosphorylation of JNK and triggering caspase activation [88]. Deletion of DLK confers robust protection from optic nerve injury [88]. In addition, genetic knockdown of DLK in human RGCs improves survival in vitro [89], indicating that human neurons are regulated by the pro-survival pathways governed by DLK. The germinal cell kinase four (GCK-IV) family was recently identified as a regulator of neuronal cell death. GCK-IV kinase inhibition not only promotes RGC survival after optic nerve crush, but, unlike DLK inhibition, actually promotes axonal regeneration [90]. 

Based on extensive work identifying critical regulators of injury-related RGC death, there are myriad intracellular pathways that could be targeted pharmacologically or transgenically to promote donor RGC graft survival following transplantation in the eye. Although manipulating neuronal survival pathways may seem like an obvious and feasible method to attenuate undesirable death of transplanted cells, we must also acknowledge the importance of these cellular pathways in driving malignant transformation. For instance, deregulation of PI3K/Akt mTOR signaling contributes to the initiation, development, and acceleration of multiple types of cancer [91]. A major contributor to ectopic malignant mTOR activation is mutations in tumor suppressor PTEN and consequent increased activity of the PI3K/Akt pathway [92]. Therefore, experimental interventions aimed at manipulating these pathways to improve donor RGC survival must include appropriate surveillance for tumorigenic activity.

Promotion of donor RGC survival by modulation of neurotrophic factor delivery may be an alternative to or compliment to directly targeting cell-intrinsic death signaling cascades. Soluble neurotrophins, such as nerve growth factor (NGF), brain-derived neurotrophic factor (BDNF), ciliary neurotrophic factor (CNTF), and glial cell line-derived neurotrophic factor (GDNF), regulate neuron growth and survival [93]. The binding of these proteins to neuronal receptors can activate intrinsic cellular signaling mechanisms and promote survival and axon growth [94] (Figure 2).

BDNF and NGF signal via tyrosine-receptor kinases (Trk) receptors to promote cell survival, or via neurotrophin receptor p75 (p75), which induces neuronal apoptosis. Indeed, the effects of NGF on RGC survival depend on the relative expression of these dueling receptors [95]. Specific activation of TrkA, but not administration of neurotrophic factors alone or even antagonization of the p75 receptor, protects against RGC death in vivo [96]. Interestingly, human iPSC-derived RGC neurite elongation in vitro is NGF responsive [97]. 

BDNF is developmentally critical to RGC survival. By binding to the TrkB receptor, BDNF triggers activation of the Ras/MEK/extracellular signal-regulated kinase (ERK) pathway [98]. Under normal physiological conditions, brain-derived BDNF supports RGC survival and synaptogenesis via retrograde transport in the optic nerve [99]. Though systemically administered BDNF does not cross the blood-brain-barrier [100], intravitreal delivery of purified protein or via AAV mediated overexpression enhances RGC survival in numerous optic nerve injury models [101,102,103]. Leveraging this neurotrophic effect, transplantation of genetically engineered MSCs that hyper-secrete BDNF protects RGCs in ocular hypertensive eyes [104]. In addition to direct signaling on RGCs, BDNF may also stimulate Müller cells and microglia to release GDNF [105]. GDNF signals directly to neurons via a heterodimer of GDNF family receptor-α and RET receptor tyrosine kinase to activate PI3-kinase and PLC-γ. Moreover, GDNF upregulates the glutamate/aspartate transporter (GLAST) in Müller glia, a process necessary to protect RGCs from toxic extracellular glutamate [106]. Long term expression of GDNF promotes photoreceptor and retinal pigment epithelial survival during retinal degeneration and RGC survival following optic nerve transection [107].

CNTF belongs to the IL-6 family of cytokines and binds a heterotrimeric membrane receptor complex composed of CNTF receptor-α, gp130, and the leukemia inhibitory factor receptor (LIF-R) [108]. CNTF promotes RGC neurite outgrowth and enhances neuronal survival and regeneration by activating protein kinase A, the Janus kinase/signal transducer and activator of transcription 3 (JAK/STAT3) and the MAPK/ERK signaling pathways [109]. CNTF is expressed in all layers of the retina, the retinal pigment epithelium, and the optic nerve head, though its expression is decreased in glaucoma patients. CNTF protects RGCs in experimental models of glaucoma and ischemic optic neuropathy [110,111]. Intravitreal CNTF protein has a short half-life [112], though AAV delivery of CNTF protects RGCs from ocular hypertension. Interestingly, the mechanism of AAV-mediated CNTF neuroprotection may be related to the immune response to treatment [113]. Sustained release of CNTF from genetically modified neural stem cells substantially decreases endogenous RGC loss for at least 4 months following axotomy [114]. Building on that progress, the NT-501 capsule containing human cells genetically engineered to hyper-secrete CNTF into the vitreous is now in phase I clinical trial for patients with primary open-angle glaucoma (ClinicalTrials.gov NCT01408472).

Given their strong potential for neuroprotection, trophic factors delivered concomitantly with RGC transplantation may substantially improve graft survival. However, clinical translation is limited by inefficient delivery of these agents. Intravitreal protein injection has a short therapeutic duration due to rapid clearance. Slow release formulations are being explored and have yielded promising results [115]. Given that certain stem cell populations, including mesenchymal stem cells, secrete high levels of neurotrophic factors [116,117], future alternative approaches may utilize mixtures of cells for delivery, or genetic modification of donor RGCs to provide sustained paracrine or autocrine delivery of neurotrophic factors, respectively [118,119]. This strategy may require concomitant efforts to sustain neurotrophic factor receptor expression [103].

### 3.3. Increasing Donor RGC Survival: Cell Extrinsic Environmental Factors

Numerous aspects of the recipient ocular microenvironment into which donor RGCs are transplanted influence their survival, including innate and adaptive immune system reactivity; blood flow, oxygen, and nutrient supply; and reactive gliosis (Figure 2).

Autologous cell transplantation is generally well accepted by the recipient, although some genetic manipulations can generate immunological responses [120]. However, successful transplantation of cells that are allogeneic, and especially xenotransplants, must overcome numerous immune system barriers involved in allorecognition to avoid graft rejection. In this regard, immunologic rejection ultimately may be a more formidable obstacle for translational studies using animal models than in eventual human clinical trials, especially if autologous iPSC-derived cells are transplanted into patients. Nonetheless, regulatory considerations may necessitate that individual iPSC transplant products undergo extensive validation and testing prior to transplantation in individual patients. If significant heterogeneity in RGC graft success from independent lines is noted, then it may be more efficient and cost-effective to develop banks of pluripotent cells lines compatible with arrays of potential recipients.

Whereas the vitreous cavity and the subretinal space are relatively immune privileged when compared to other sites, damage to the blood ocular barrier and local inflammatory cytokine production can be induced by both neurodegenerative disease and the transplantation procedure itself [121]. Intraocular cell transplantation is subject to chronic immune system activation and graft rejection, where variation in a single amino acid between the donor and the recipient protein sequence is enough to prompt destruction of the transplant [122,123]. When foreign cells are recognized, alloreactive cytotoxic CD8^+^ T cells are activated through the binding of MHC class I and T cell receptors, particularly in the presence of non-matching major histocompatibility complex (MHC; known in humans as human leukocyte antigen, HLA) [124,125,126]. For both experimental purposes and eventual clinical translation, a greater understanding of immunosuppressive requirements for RGC transplantation is needed. Xenotransplantation in rodents has typically utilized systemic immunosuppression with various combinations of cyclosporine-A, azathioprine, and/or prednisone [34,127].

Editing of individual HLA genes in iPSCs to establish “universal donor cells” has been proposed for evasion of both T cell activity caused by HLA mismatching and natural killer (NK) cell activity. Genetic manipulation, such as the transgenic expression of HLA-E or HLA-G on porcine endothelial cells inhibits NK cell cytotoxicity and adhesion [128]. Genetically modifying stem cells to successfully evade T cell and NK cells is a feasible means of protecting against xenotransplant rejection [129]; however, additional mild immunosuppressants can still be useful [129,130]. Thus, better understanding how to achieve immune tolerance will provide a means of avoiding rejection and improving survival in the transplantation of stem cell derived RGCs. 

The transplantation technique itself is critical to consider when evaluating factors that might affect transplanted RGC survival. To date, the majority of studies have injected cells directly into the vitreous cavity, where the supply of oxygen and nutrients is subject to passive diffusion from the uveal vasculature. During development, the lens and the developing inner retina are first vascularized by the hyaloid canal, composed of the hyaloid artery, vasa hyaloidea propria, and tunica vasculosa lenti, which lie within the vitreous cavity. However, at birth in humans and postnatally in mice, these vessels regress as retinal vessels are prioritized, leaving the vitreous cavity unperfused [131]. In order to achieve successful transplantation and survival or RGCs, it may be necessary to support RGCs metabolically until they reach the retina, or to transplant RGCs into direct proximity with the host retina, potentially within a scaffold. 

It is critical to recognize that RGC transplantation will only be necessary because a primary neurodegenerative process caused extensive death of endogenous RGCs. Glaucoma, optic neuritis, and ischemic optic neuropathy are the most common neurodegenerative diseases characterized by RGC loss, but each one has unique pathophysiologies [132,133]. Once integrated into the visual pathway, one must assume that, in most cases, donor RGCs will be subject to the same pathogenic insults and stressors that created therapeutic necessity. However, ongoing neurotoxic stress may not be uniform for all optic neuropathies. Acquired toxic or nutritional optic neuropathies, for instance, might be amenable to removal of the inciting agent and are therefore associated with a more receptive microenvironment. Hereditary conditions, such as Leber optic neuropathy or autosomal dominant optic atrophy, are candidates for gene therapy correction of the donor RGCs [134] if autologous cells are transplanted. To the extent that RGC death itself creates secondary neurotoxic effects, however, any therapeutic RGC transplant may face environmental challenges. Therefore, understanding the neuropathologic mediators that contribute to multiple forms of RGC injury may aid development of approaches to enhance donor RGC survival. While the importance of disease-specific mechanisms of RGC death will undoubtedly also be important, they are outside the scope of this review.

Innate neuroinflammatory processes influence donor RGC survival following injury and are principally mediated by retinal macroglia (astrocytes and Müller glia) and microglia. Under normal conditions, astrocytes play a key role in maintaining homeostasis in the CNS, controlling angiogenesis, contributing to the extracellular matrix (ECM), and maintaining the blood retinal barrier. Upon disruption or injury, reactive glia undergo complex alterations in gene expression, morphology, and function. Reactive astrocytes are promoted by JAK/STAT signaling, which leads to the release of a variety of soluble mediators that activate inflammation within the CNS [135,136]. Historically, this was considered primarily detrimental by leading to glial scar formation and inhibiting regeneration [137]. These changes are driven by molecular triggers, including proinflammatory cytokines, tumor necrosis factor-α (TNF-α), hypoxia, oxidative stress, and nitric oxide (NO) production [138]. However, emerging evidence suggests that, on a cell population level, glial responses to neurodegeneration may be either neurotoxic or neuroprotective. A1 neurotoxic astrocytes are induced by activated microglia through secretion of TNFα, Il-1α, and C1q. They are rapidly activated after acute CNS injury and produce marked neurotoxic effects in co-culture with RGCs, presumably by secretion of, as of yet unidentified, toxic factors. A2 astrocytes are thought to promote neuronal survival and repair, in part by upregulating neurotrophic factor production [139]. In a mouse model of glaucoma, ocular hypertension induction is sufficient to trigger the production of cytokines necessary to drive the formation of A1 astrocytes. Genetic deletion of TNFα, Il-1α, and C1q, or treatment with a glucagon-like peptide-1 receptor (GLP-1) agonist, results in the reduction of A1 astrocytic activity and amelioration of RGC death [140]. Importantly, the functionality assignment of these reactive glial phenotypes is based on correlative evidence in bulk cell analyses. Causation-testing, including loss or gain of function experiments, and glial phenotyping at a single cell level have yet to definitely link genetic markers, signaling triggers, and effector molecules to either the “toxic” or “protective” phenotype [141].

Some of the effectors of neuroinflammatory toxicity have been elucidated. In response to CNS injury or neurodegenerative disease, multiple cell types produce cyclooxygenase-2 (COX-2), leading to prostaglandin E_2_ (PGE2) synthesis, which contributes to neuronal death [142]. Anti-inflammatory and anti-oxidant agents, such as resveratrol, are neuroprotective in retinal ischemic injury and glaucoma [143]. Glial and immune cells are major producers of oxidative stress mediators, such as endothelial nitric oxide synthase (eNOS), inducible nitric oxide synthase (iNOS), and NO. Oxidative stress is often viewed as being contributory to neuronal death; however, it can, at times, be neuroprotective, for instance as a vasodilator maintaining perfusion in ischemic retinas [144,145]. Nonetheless, NO increases intracellular Ca^2+^ concentrations and induces CREB-mediated transcription of apoptotic proteins, resulting in cell death [146]. Thus, neuroinflammation and oxidative stress may be primary targets of environmental modulation to promote donor RGC survival following transplantation.

## 4. Transplanted RGC Migration and Somal Integration

### 4.1. Assessing Donor RGC Laminar Localization

Donor cells arriving at the retina from the vitreous cavity encounter the retinal basement membrane (the ILM), local astrocytic and Müller glial reactivity, and local inflammatory cells that impede spontaneous cell migration into the retina [16,147]. Endogenous molecules from the extracellular matrix contribute to the migratory blockade of transplanted cells. In fact, degradation of chondroitin sulfate proteoglycans (CSPGs), which are extracellular matrix components involved in cell adhesion and growth, promotes the intraretinal migration of transplanted mesenchymal stem cells [147]. The deposition of this extracellular matrix protein is inhibitory to the formation of new synaptic connections, and an inherent protective mechanism of the mammalian CNS to impede aberrant neuronal synapses after injury [148]. On the other hand, peeling of the ILM enhances migration of intravitreally transplanted cells into the retina. Interestingly the mechanism of this effect in MSCs depends not on removal of the basement membrane per se, but on damage to Müller glial endfeet and suppression of reactive gliosis. Even a transient decrease in reactive gliosis in vivo significantly augments entrance of MSCs into the retina [149]. 

**Figure 3:**
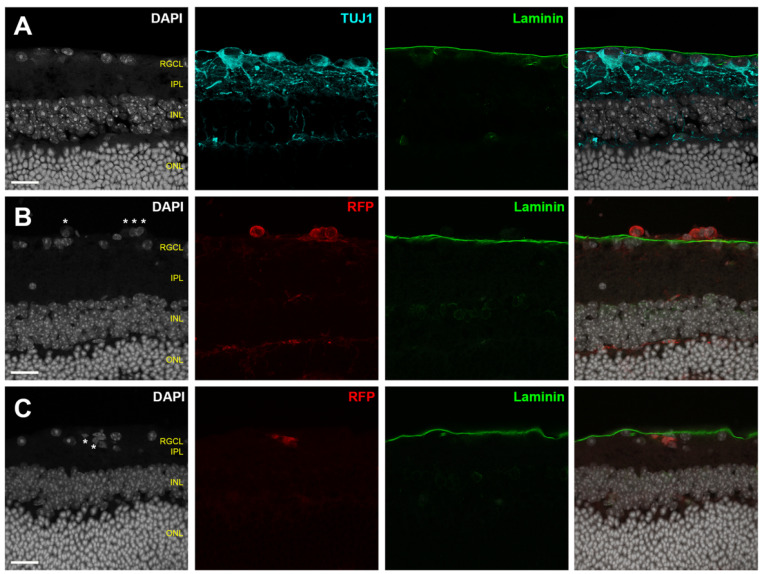
Adult mouse retinal cross-sections showing laminin expression (green) in the intact ILM in relation to endogenous TUJ1^+^ RGCs (row **A**, cyan) and intravitreally transplanted RFP^+^ human stem cell derived RGCs (**B**,**C**, red). DAPI staining (white) demonstrates close proximity of transplanted RGC (asterisks) to the endogenous RGCs. Endogenous RGCs reside within the retinal parenchyma bounded by the ILM (**A**). Transplanted RGCs are typically excluded from the host RGCL (**B**), but rarely may migrate across an intact ILM and localize within the host retinal parenchyma (**C**). Scalebars: 20 μm. RGCL—retinal ganglion cell layer; IPL—inner plexiform layer; INL—inner nuclear layer; ONL—outer nuclear layer.

The ability of transplanted RGCs to migrate spontaneously into the recipient retina is inconsistent across published studies, which may be related to differences in graft and host species, or to the methodologies of determining graft localization. Our previous ex vivo work has identified that the ILM is a major barrier to both human RGC somal and neurite engraftment; we have found that donor human RGC migration into the host mouse retinal parenchyma rarely occurs spontaneously [16]. Disrupting ILM integrity can profoundly reverse donor sequestration outside the retinal parenchyma. Intravitreally injected mouse iPSC-RGCs are reported not to engraft into healthy host retina, and instead aggregate along the vitreoretinal interface [53]. Similarly, Muller glial-derived RGCs accumulate on the vitreous side of the ILM without migrating through an intact ILM [150,151]. These studies do not conclude evidence of donor cell integration into the host RGCL. In contrast, several in vivo studies have interpreted donor cell localization as representative of spontaneous host RGCL localization. Transplanted primary mouse RGCs localize to the RGCL without ILM disruption, albeit at very low efficiency [32]. Intravitreally injected spermatogonial stem cell (SSC) derived RGCs are found in close proximity to host RGCs 10 days after transplantation [74]. hESC-RPCs transplanted into an excitotoxicity mouse model of retinal damage identified donor cells near host RGCs [72]. However, we note that it can be challenging to know based on proximity alone whether a donor cell is truly located within the retinal parenchyma or just on the external side of the ILM (Figure 3). Within the published literature, standardized metrics for evaluating donor cell coplanarity in the RGCL are lacking. Furthermore, there is a paucity of data quantifying the rate at which such structural integration occurs.

Available evidence supporting donor integration is typically derived from en face confocal micrographs illustrating close proximity between donor cells and host RGCs, or from histological sections, again relying on proximity to the endogenous RGCs. However, such demonstration typically lacks the spatial resolution to distinguish the anatomical layers at the vitreoretinal interface. Because the RGCL is in close proximity to the ILM, donor RGCs that are external to the retinal parenchyma (within the posterior vitreous cavity) but juxtaposed to the retina can easily be mistaken for having “integrated” into the retina, both in retinal flat mounts and in histological sections (Figure 3). Therefore, reporting donor integration requires careful examination, and developing methods to overcome integration barriers and to validate donor coplanarity with the host RGCs is critical to cell replacement therapies.

**Figure 4:**
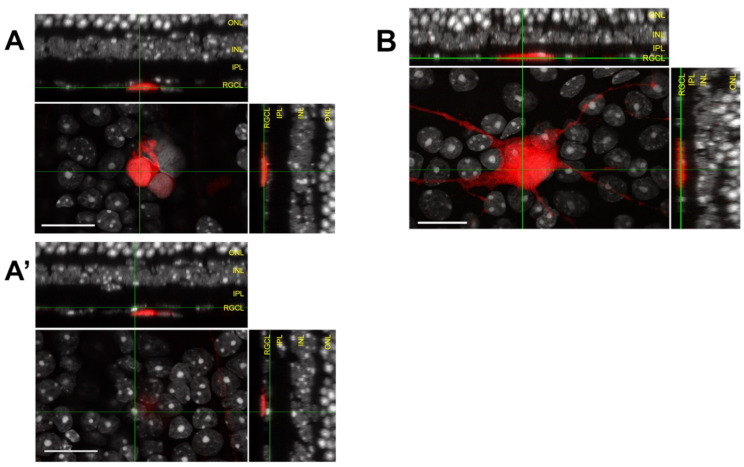
Orthogonal views from the same confocal microscope z-stack of two different planes (**A**,**A’**) used to analyze relative planarity of donor RGC (red) and endogenous RGCs (grey). Donor RGC in (**A**) is situated in a superficial *z* plane as compared to the endogenous RGCs (**A’**) underlying the same *xy* position. Example of a coplanar donor RGC (red) in (**B**) localizing to the endogenous RGCL, as indicated by the orthogonal views showing total coplanarity between the donor soma and nuclei of surrounding endogenous RGCs. Scalebars: 20 μm. RGCL—retinal ganglion cell layer; IPL—inner plexiform layer; INL—inner nuclear layer; ONL—outer nuclear layer.

Although retinal cross sections can clearly demonstrate the relationship between transplanted RGCs and endogenous RGCL, applying this method to completely survey entire eyes is unfeasible. To help meet this challenge, we propose the following considerations for microscopic inspection of donor RGC localization within the host retina (Figure 4):

- A combination of analyses using histological sections and en face evaluation of retinal flatmounts will provide a compromise between complete topographic imaging and depth resolution;
- Flatmount microscopy should be performed with high resolution confocal or multiphoton microscopy using a high magnification objective in order to create shallow depth of field;
- High depth resolution is attained by minimizing slice interval distance and pinhole aperture diameter (if single photon microscopy is employed);
- Z-stack reconstructions should be assessed using an orthogonal slice viewer to compare the localization of the donor cell to those of nearby endogenous cells;
- A secondary marker that outlines the boundary of the retinal parenchyma should be included when possible, such as immunofluorescent laminin delineation of the ILM (Figure 3).

Scrutinizing donor RGC integration using this robust approach sets a benchmark for accurate assessment of donor cell localization. The reported outcomes from the localization analyses should include the absolute number and percentage of total surviving RGCs in each of the retinal layers (RGCL, IPL, INL, ONL) as well as outside of the retinal parenchyma. Somal localization is important because donor RGCs with cell bodies remaining outside of the host retina can rarely exhibit parenchymal neurite ingrowth, which may be distinguished from fully integrated donor cells.

In our experience, we observe negligible spontaneous donor human RGC integration into the host RGCL when the ILM remains intact, whereas somal integration is greatly enhanced by enzymatic disruption of ILM [16], suggesting the inner retinal extracellular matrix is a significant structural barrier to transplanted RGCs integration. A chronic progressive mouse model of glaucoma demonstrated pathologic retinal ECM remodeling and upregulation of several families of glycoproteins, including laminin and fibronectin [152]. Together with evidence that ECM alterations impact endogenous RGC survival during optic neuropathy disease progression [153], the interactions between ECM and donor RGCs for both survival and migration must be considered. Future RGC transplantation studies in disease models should examine the impact of ECM remodeling on donor integration. For example, in addition to transplanting into healthy eyes, experimental transplantation should include acute and chronic glaucomatous recipients where the extracellular milieu is known to undergo significant alterations. 

### 4.2. Assessing Donor RGC Retinotopic Mosaicism

We have observed a propensity for transplanted RGCs to cluster when cultured on organotypic retinal explants with intact ILM [16]. Proteolytic ILM digestion eliminates cell clumping and achieves greater donor cell coverage of the retinal surface. The mechanism driving topographical distributions of transplanted RGCs is unclear, but our observations suggest that interactions between donor RGCs and components of the ILM promote cell migration and clustering. Interestingly, tangential migration across the retina rarely occurs by endogenous RGCs during development [154], and developing RGCs halt migration once axonogenesis is initiated [155]. These phenomena may be related to the fact that RGCs require stable receptive fields in a mosaic pattern and minimal dendritic overlap with neighboring RGCs. Therefore, attaining widespread retinal coverage by transplanted RGCs will either require migratory cell behavior not typical of development, or will require methodologies of introducing RGCs in a dispersed fashion. Otherwise, transplanted RGCs may compete with each other for pre-synaptic partners, or fail to adequately sample the visual field. 

Considering this challenge, studies of RGC transplantation should include quantitative characterizations of cellular dispersion and retinal coverage. We have employed spatial analytic tools to describe the topography of donor RGCs cultured on the retinal surface ex vivo [16], and these quantifications may be translated easily to in vivo transplantation studies. Metrics such as nearest neighbor distance (NND), nearest neighbor index (NNI), density recovery profile (DRP), and Ripley functions collectively paint a descriptive picture of the two-dimensional topographic donor cell distribution pattern and, critically, allow for quantitative comparisons between experimental groups and independent studies. These statistical metrics have been used previously to describe mosaic patterns of endogenous retinal neurons, and best practices for employing these tools have been elegantly reviewed [156]. Attaining donor RGC topographical distributions that resemble those of endogenous RGCs, either by altering donor-host interactions though cell surface receptor expression or by modulating RGC intrinsic pathways to promote lateral spreading, will be necessary to achieve widespread retinotopic coverage.

## 5. Transplanted RGC Dendritogenesis and Afferent Synaptogenesis

### 5.1. Assessing Donor RGC Neurite Localization

After overcoming barriers to localization of transplanted RGCs within the recipient retina, dendrite targeting of the IPL necessarily precedes synaptogenesis to achieve true functional integration into the visual neurocircuitry. Donor RGCs are unlikely to sprout neurites without specific external cues [157]. One such signal during development is laminin within the ILM [158]. Absence of laminin contact during development leads to RGC mispolarization, and laminin supplementation in vitro is sufficient to induce neurite growth [158]. Disruption of RGC recognition of laminin also alters RGCL patterning during development [159]. Without disrupting the ILM in adult rodent recipients, only a small fraction of transplanted mouse RGCs into healthy rodent hosts are able to localize to host retina, but those that do are able to extend axons toward the optic nerve head and dendrites into the IPL [32]. In contrast, most transplanted RGCs do not exhibit distant axon extensions [34,71,74], possibly because they do not localize to the inner retinal parenchyma. Only a subset of published transplantation studies report evidence of donor cell neuritogenesis [32,33,62,70,73,74], and even fewer have described dendritic lamination into the host IPL [32,62]. In light of this, maintaining the ILM as a neuritogenic signal and growth substrate may be important, and enzymatic digestion or mechanical peeling to encourage RGC somas to enter the retinal parenchyma may actually undermine subsequent steps in neural integration [160]. Methods to transiently overcome RGC interactions with the ILM during migration may, therefore, be useful.

The proper targeting of donor cell dendrites to the mature host IPL is an understudied aspect of RGC transplantation. Developmental studies reveal that RGC dendritic lamination in the IPL is guided by signaling through cell surface receptors, and dendrite localization is reinforced and stabilized by neuronal activity [161]. RGCs utilize subtype-specific cell adhesion molecules to pattern IPL sublaminar stratification, some of which include integrins, cadherins, and plexins [159,162,163]. Tbr1, a transcriptional regulator of Cadherin 8, is sufficient to specify dendrites from multiple OFF-laminating RGC subtypes to the outer IPL [164]. Ectopic expression of Trb1 in neonatal mouse retinas directs OFF-RGCs to extend dendrites to the outer IPL. RGCs ramifying in both the ON and OFF substrata of the IPL selectively express Satb1, which is necessary to express the adhesion molecule Contactin 5, and which is involved in bistratified IPL patterning [165]. Members of the immunoglobulin superfamily adhesion molecules, including DSCAM and Sidekick, are expressed in specific RGC subtypes to establish sublaminar specificity of their synapses [166]. Knocking down DSCAM and Sidekick expression results in disrupted dendritic patterning, and their ectopic expressions are sufficient to re-direct laminar patterning in vivo. However, it is unknown whether critical RGC-extrinsic dendritic localization signals remain expressed in the mature and/or diseased retina to facilitate afferent targeting of donor neurites, or if genetic regulators governing laminar specification need to be modulated in transplanted RGCs or the recipient microenvironment. Even if so, the recognition of those signals by donor RGCs will likely depend on RGC subtype specification.

**Figure 5:**
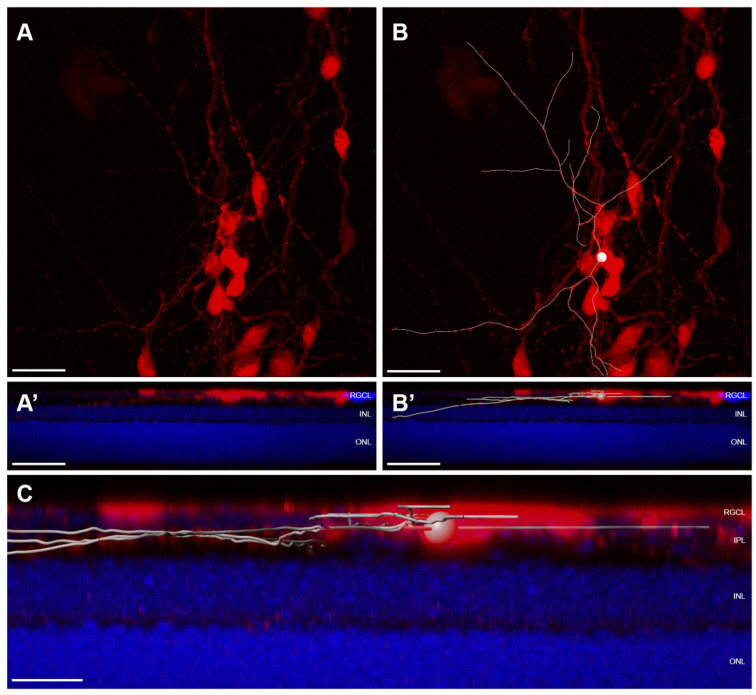
Three-dimensional reconstruction of confocal microscopy showing in vivo transplanted hESC-RGCs (red) in pronase-treated wildtype mouse retinas. En face projections of donor RGC soma and neurite growth (**A**) and tracings of integrated neurites from a single cell in white (**B**). Integrated RGC soma is indicated by the sphere (**B**,**B’**,**C**). DAPI (blue) demarks retinal layers in orthogonal projections of the confocal z-stacks (**A’**,**B’**,**C**). Scalebars: 30 μm (**A**,**A’**,**B**,**B’**); 10 μm (**C**). RGCL—retinal ganglion cell layer; IPL—inner plexiform layer; INL—inner nuclear layer; ONL—outer nuclear layer.

In order to robustly characterize the dendritic outgrowth of donor RGCs, we raise the following considerations:

- Retinal flatmounts are superior to histological section for characterizing complex dendritic arbors of RGCs within the IPL;
- Additional secondary tissue samples for histological sectioning can, however, be useful for correlation of sublaminar neurite localization with multiple immunohistochemical markers that define RGC subtype;
- It is of interest to characterize the topographical spacing among integrated RGCs. Reported metrics may include nearest neighbor and density recovery profiles of integrated donor RGCs;
- When possible, metrics describing dendritic architecture should be described for individual cells; if individual arbors are not resolvable from overlapping RGCs, then metrics should be normalized to the number of donor RGCs contained within a region of interest;
- Dendritic architecture and localization within the recipient retina should be assessed using high resolution microscopy; it may be necessary to tile images in order to capture entire dendritic arbors at high resolution;
- Integrated dendritic arbors can be convoluted and should be resolved and traced using 3D rendering software (Figure 5);
- Useful metrics to describe dendritic arbors include total neurite length, number of neurite segments, neurite density, dendritic Sholl analysis [167], and neurite distribution in each retinal layer, and where applicable, within specific IPL sublamina.

If neurite integration is to be evaluated, detailed characterization of neurite identity will be needed. In mature RGCs, dendrites and axons are defined by the expression of MAP2 and Tau, respectively [168]. Neurites in *developing* RGCs express both MAP2 and Tau until definitive specification of an axon [169]. Therefore, donor RGCs may colocalize MAP2 and Tau in early neurite growth, and the degree of segregation in MAP2 and Tau expressions may be an indication of RGC maturity. We have found that 90% of donor RGCs that have not integrated into the retinal parenchyma exhibit neurites that co-express MAP2 and Tau, and structural localization of donor RGCs to the retina is associated with an increased proportion of neurites expressing only MAP2 or only Tau [16]. This finding may suggest structural integration of donor RGCs into the host retina influences neurite specification.

### 5.2. Assessing Donor RGC Functional Connectivity within the Recipient Retinal Neurocircuitry

There are over 40 subtypes of RGCs, each having dendritic arbors precisely restricted to specific lamina within the IPL [170]. Such heterogeneity creates challenges in defining the normal RGC connectome, which is amplified when considering integration of transplanted RGCs into an existing pathological neurocircuitry. Convincing evidence of functional synaptogenesis following RGC transplantation is scarce and should be a priority of ongoing work. Immunohistochemical detection of synaptic machinery localizing to donor RGC processes [32,73] or whole cell electrophysiological recordings [32] provide some evidence of afferent connectivity of transplanted RGCs. However, there are significant limitations to these approaches. Immunohistochemical demonstration of colocalizing pre- and postsynaptic markers lacks specificity because endogenous synapses far outnumber the synapses on donor neurites and may lead to false colocalization. Electrophysiological approaches are useful to identify specific examples of true functional integration based on the presence of light evoked (photoreceptor transduced) post-synaptic potentials, but the ability to assess very large numbers of donor RGCs is hampered by feasibility and throughput. Thus, alternative approaches to assess neural connectivity must be reliably performed on a large scale so as to provide rigorous quantification of functional integration. Suitable methods may include (1) transsynaptic circuit tracing and/or (2) optical electrophysiology with genetically encoded fluorescent indicator proteins.

Viral transsynaptic tracers make it possible to decipher complex circuit connectivity. Individual viruses have defined directionalities of travel and benefit from intracellular replication that propagates reporter signal generation [171]. The most common viruses used for neural circuit tracing include the H129 strain of herpes simplex virus type 1 (HSV-1), Bartha pseudorabies virus (PRV), rabies virus, and adenovirus serotype 1 (AAV-1). Among these, H129 and AAV-1 travel in the anterograde direction [172,173], while PRV and rabies exhibit retrograde spread. Beyond the property of directionality, the Bartha PRV can cross multiple synapses [174,175], whereas AAV-1 restricts the spread to only the first-order postsynaptic neuron [173]. While initially used to trace endogenous anatomic neural circuits, viral transsynaptic circuit tracing is being employed in neuronal transplantation, as recently elegantly reviewed [176]. A significant limitation of this approach, however, is that viral infection of individual neurons can produce marked cytotoxic effects and so the utility of this technique tends to be limited to anatomic tracing, short time courses, and important caveats when studying the cell physiology of labeled cells.

There are numerous paradigms available to restrict uncontrolled propagation of virus across multiple synapses, to specify neuronal subtypes for primary infection (starter cells), and to not only label but also genetically modify synaptically coupled partner neurons through payload packaging. Typically, viruses used for synaptic circuit tracing are modified and incapable of infectivity and/or replication without helper genes (i.e., envelope glycoprotein G and EnvA for Rabies virus, or thymidine kinase for HSV) that are supplied to starter neurons separately. Through the use of Cre-dependent helper gene expression in conjunction with Cre reporter lines, one could, for instance, restrict starter cells to retinal neuronal subtypes of interest and probe donor RGCs for evidence of viral transsynaptic spread following transplantation [177]. A comparable approach was recently utilized to catalog dopaminergic neuronal graft integration in a mouse model of Parkinson’s disease [178].

Large scale visualization of neuronal activity can be achieved by imaging genetically encoded fluorescent indicator proteins. Various optical reporters have been devised in response to vesicle release, membrane voltage change [179], and calcium ion flux [180]. In particular, genetically encoded voltage indicators and calcium indicators have been widely used to provide fluorescent readouts of neuronal electrophysiological activity. Incorporating these genetically encoded optical indicators in donor RGCs and imaging their fluorescent signal after light stimulation can provide a reliable way to screen for functional connectivity with the host retina. However, the fact that fluorophore excitation requires visible light exposure to the retinal flatmount and evokes visible light emission from the reporter protein means that the act of imaging the indicator will also provide light stimulation to the photoreceptors. This confounds the measurement of light-evoked electrophysiologic activity in donor RGCs, but may be at least partially circumvented by using two-photon infrared excitation.

Electrophysiology and synaptic histology remain useful for characterizing specific examples of functional synapses. Whole cell patch clamping of donor RGCs facilitates recording of light-evoked electrophysiological responses in retinal flatmount preparations. So long as controls are included to exclude the possibility of recording from intrinsically photosensitive RGCs, electrophysiologic responsivity to light implies stimulus conduction from bipolar cells downstream of photoreceptor transduction. Characterization of specific responses to light permits classification of donor RGCs based on electrophysiological properties.

Synapses in retinal neurons are unique in that they require rapid and sustained release of neurotransmitters in order to achieve graded synaptic output in response to a dynamic range of light signal intensity. Consequently, retinal synapses require a several fold higher number of vesicles than conventional synapses that transmit action potentials elsewhere [181]. Synaptic terminals found in bipolar cells form “ribbon synapses”, where a large pool of vesicles is tethered to a linear structure in the shape of a ribbon. The major protein constituents of bipolar cell ribbon synapses are RIBEYE proteins that build the ribbon scaffold, and Piccolo that anchors the ribbon to the presynaptic membrane [182,183]. Additionally, synaptophysin and synaptoporin are the most abundant synaptic vesicle proteins in the IPL [184]. Aggregated synapse-associated proteins can be visualized by immunohistochemistry as punctate appearance, while the electron dense linear ribbon can be viewed on TEM [185]. Immunolabeling and colocalization of presynaptic markers with PSD95, the postsynaptic scaffolding protein, is suggestive of synaptic pairing in RGCs.

These techniques may prove useful in future transplantation studies examining factors that regulate synaptogenesis between donor RGCs and the mature recipient retinal neurons. Synthetic synaptic organizers have been developed that are capable of inducing functional excitatory synapses within the CNS [186]. Their function in constructing novel circuits built through transplantation might be assessed as one possible method to bridge transplanted RGCs with host retina.

## 6. Material Transfer

One major consideration that is required for all transplantation studies is the possibility of intercellular material exchange [187,188,189,190], a phenomenon that could lead to misinterpretation of donor cell survival and integration. This confounding phenomenon was first identified in photoreceptor transplantation, where donor cell fluorescent reporter signals observed in the host recipient outer nuclear layer were initially thought to signify physical donor photoreceptor integration. It is now understood that intercellular transfer of fluorescent reporters from the donor to host photoreceptors accounted for a large proportion of the initially presumed “engraftment”. Extensive analyses, including using mismatched donor-host fluorescent reporters, fluorescent in situ hybridization of X and Y chromosomes in sex-mismatched donors and hosts, Cre-recombinase reporter paradigms, and EdU labeling of donor cell nucleic acids, have all concluded that many fluorescently labeled cells in the host ONL using legacy experimental techniques were host-derived [187,188,189,190]. This discovery has prompted close scrutiny of photoreceptor transplantation results since 2016, and should similarly be applied to RGC transplantation where a standard set of labeling tools and cell lineage markers can help ensure reliable interpretation of donor RGC integration.

## 7. Safety Considerations

Using human stem cells in RGC transplantation requires balancing safety and efficacy against potential risks and ethical considerations. Ethics concerns surrounding ESC use has largely been avoided with iPSCs as an alternative stem cell source. However, colonies expanded from a single iPSC display more heterogenous gene expression compared to ESCs [191], elevating concerns regarding the proliferative potential of the donor cells causing teratogenicity [192]. Thus, pluripotency and mitotic activity must be monitored after engraftment and can be addressed by selectively identifying and eliminating undifferentiated pluripotent cells through chemical treatment [193]. Additionally, allogenic MSC transplantation has been documented to cause hazards to the recipient [194,195]. Perturbed visual function, retinal detachment [196], RGC loss, and venous congestion have been reported as a result of transplanted MSCs triggering intraocular inflammation [197], and they may be a concern for other cell types as well. Furthermore, stem cell derived RGCs may differ from native RGCs in unexpected ways. Therefore, full characterizations of donor cells using genomic, transcriptomic, and proteomic approaches are needed prior to trialing this approach clinically.

## 8. Future Directions

Recent scientific advances have enabled RGC production from a wealth of human stem cell sources for both disease modeling and RGC replacement within the visual pathway. Developments in stem cell biology and regenerative neuroscience have brought the field of optic nerve regeneration from science fiction to scientific possibility. Nonetheless, tremendous challenges for translating this potential to actual vision restoration for human patients lie ahead. Improving transplant survival remains the primary short-term goal, as it sets the foundation for subsequent study of functional integration. Advanced differentiation protocols may provide reliable RGC subtype specification and expand the donor source repertoire. As imaging techniques continue to improve, we anticipate significant progress in the ability to assess migration, localization, synaptogenesis, and axonogenesis in vivo.

It is not well established how closely transplanted human stem cell derived RGCs resemble developing or mature neurons. This distinction may be important because the molecular machinery governing growth and regeneration potentially involves separate sets of regulatory mechanisms [198]. For example, axonal growth in immature neurons precedes synaptogenesis with central targets, after which the neuron transitions away from a growth state to a synaptogenic state. Damaged mature neurons remain in the synaptogenic mode by default, which could result in ectopic synaptogenesis at the injury site [198]. Thus, an in-depth understanding and careful fine-tuning of intrinsic and extrinsic pathways affecting donor RGCs are required to promote visual pathway connectivity, while minimizing haphazard targeting.

## Figures and Tables

**Table 1:** Summary of RGC sources for experimental, and potentially clinical, transplantation.

Donor Cell Source	Advantages	Disadvantages
Primary murine RGCs	Compatible allogeneic transplantation in murine recipeints Bona fide RGCs based on normal development	Limited scalability Limited/no clinical potential
Stem cell-derived murine RGCs	Autologous or allogenic transplantation in murine hosts Multiple published differentiation protocols Scaleable and renewable	Potential teratogenicity Limited/no clinical potential
Human ESC-derived RGCs	Scalable and renewable Multiple published differentiation protocols Translational potential	Ethical concerns Potential teratogenicity Line-to-line heterogenity Finite number of parental lines; limitations in establishing new lines
Human iPSC-derived RGCs	Scalable and renewable Multiple published differentiation protocols Potential for autologous transplantation Unlimited ability to establish new and specialized cell lines Translational potential	Line-to-line heterogenity Potential teratogenicity

**Table 2:** Summary of published experimental RGC transplantation studies.

Donor Cell Source	Host Species	Disease Model	Injection Route	Injection Vehicle	Immuno-Suppressive Regiment	# of Donor Cells in Transplant	Experiment Duration	Presence of Donor Cells in Host Eyes	RGC Survival Rate	Host Retina Localization	Neurite Formation	Functional Improvement	Ref
hESC-RPCs	NHP	Healthy eyes	Subretinal	Media (DMEM/F12, N2, B27, NEAA, pen/strep, DAPT)	None	1 × 10^6^/eye	1–3 months	Yes	Not reported	RGCL, INL	Yes (Projections toward ONH)	Not reported	Chao et al. (2017) [70]
hESC-RGCs	Rat	Healthy eyes	Intravitreal	Media (Sato medium containing DAPT)	None	5 × 10^4^/eye	1 week	Yes (5/5 eyes w/ detectable donor cells)	19–25 cells/mm^2^	RGCL (HuNu^+^ RGCs near Tuj1^+^ retinal layer)	Not reported	Not reported	Zhang et al. (2020) [71]
hESC-RPCs	Mouse	NMDA excito-toxicity	Intravitreal	Media (DMEM/F12, N2, KOSR, L-glutamine, non-essential amino acids, nicotinamide)	None	2 × 10^4^/eye	4–5 weeks	Yes	Not reported	RGCL (HuNu^+^ donor cells near Brn3a^+^ host cells)	Not reported	Not reported	Wang et al. (2019) [72]
hiPSC-RGCs	Rabbit, Monkey	Healthy eyes	Intravitreal	PLGA scaffold	None	1 × 10^5^/scaffold	1 week–3 months	Yes	Not reported	Not reported	Yes (RGCs on scaffolds form dendrites; express Neuro-filament)	Not reported (Donor RGCs express voltage-gated Na^+^ channels)	Li et al. (2017) [73]
hiPSC-RGCs	Mouse	ONC	Intravitreal	MACS buffer	Cyclosporine (210 mg/L) in drinking water	2 × 10^5^/eye	1–4 weeks	Yes (10 of 17 eyes)	Not reported	RGCL (9/17 eyes showed donor cells in close proximity to host RGCL)	No	Not reported	Rabesandratana et al. (2020) [34]
hSSC-RGCs	Mouse	NMDAexcito-toxicity	Intravitreal	FACS buffer	None	1 × 10^4^/eye	10 days	Yes (unspecified fraction of eyes demonstrated survival)	Not reported	RGCL (donor cells found nearby endogenous RGCs)	No	Not reported	Suen et al. (2019) [74]
rRGCs	Rat	Healthy eyes	Intravitreal	PBS	None	5 × 10^4^/eye	1–7 days	Yes	~3% on day 1 ~1% on day 7	NFL (donor cells along the host Tuj1^+^ NFL) RGCL (proportion of donor cells intermingled w/ host RGCL)	No (In vivo: not reported; ex vivo ~75% neurite outgrowth in developing RGCs, ~20% in adult donor RGCs)	Not reported	Hertz et al. (2014) [75]
mRGCs	Rat	Healthy eyes	Intravitreal	Media (Neurobasal, insulin, pyruvate, L-glutamine, T_3_, NAC, GS21, BDNF, CNTF, forskolin)	None	4-6 × 10^4^/eye	1–4 weeks	Yes (15 of 152 eyes)	<1–7%	RGCL (15/152 eyes)	Yes (>90% of surviving cells, some of complex morphology)	Yes (PSD95^+^ donor neurites; light-evoked postsynaptic current)	Venugopalan et al. (2016) [32]
mRGCs	Rat	Healthy eyes	Intravitreal	Cotransplantation w/ hiPSCs in media (StemMACS iPS-Brew XF, Miltenyi Biotec)	None	4 × 10^4^/eye	1 week	Yes (20% of experiments w/ retinal engraftment)	<1% increasing to approx. 3.5% with iPSC co-transplant	Not reported	Yes (Increased by hIPSC co-transplant)	Not reported	Wu et al. (2018) [76]
mESC-RPCs	Mouse	NMDA- excito-toxicity	Intravitreal	PBS with 10 ng/mL FGF2	None	1 × 10^6^/eye	2 months	Yes	Not reported	RGCL (flat mount and sectioned retina w/ GFP^+^ donor cells near host RGCL)	Yes (GFP^+^ cells w/ neurite morphology resembling endogenous RGC)	Yes (optokinetic tracking and light avoidance, c-Fos expression)	Divya et al. (2017) [33]
		DBA/2J mice	Intravitreal		None	1 × 10^6^/eye	2 months	Not reported	Not reported	Not reported	Not reported	No improved visual acuity	
miPSC/mESC-RGCs	Mouse	Healthy eyes	(1) Intravitreal; (2) Subretinal	Media (DMEM, Glutamax, non-essential amino acids, pyruvate, lipid concentrate, antibiotics, b-mercaptoethanol, NS21, NAC)	None	2 × 10^4^/eye (adult recipient); 1 × 10^4^/eye (early postnatal recipient)	2 weeks–12 months	Yes (At 2 weeks: 8/10 adults; 9/9 pups. At 12 months: 2/4 mice)	0.5–5%	RGCL (Thy1-GFP^+^ donor cells adjacent to host RGCL) ONH (some donor cells migrated into the nerve head)	Yes (diverse morphology, ranging from no neurite to laminated processes)	Not reported, but evidence of synaptic connection with host by WGA tracing	Oswald et al. (2021) [62]
		Microbead-induced high IOP;	Intravitreal		None	2 × 10^4^/eye	2 weeks	Yes (4/6 mice)	0.1–1%	Not reported	Yes (~50% of cells formed neurites)	Not reported	
		NMDAexcito-toxicity	Intravitreal		None	2 × 10^4^/eye	2 weeks	Yes (4/6 mice)	0.1–1%	Not reported	Yes (~25% of cells formed neurites)	Not reported	

hESC-RGCs—human embryonic stem cell derived RGCs; hESC-RPCs—human embryonic stem cell derived retinal progenitor cells; hiPSC-RGCs—human induced pluripotent stem cell derived RGCs; hSSC-RGCs—human spermatogonial stem cell derived RGCs; mRGCs—purified primary mouse RGCs; miPSC-RGCs—mouse induced pluripotent stem cell derived RGCs; mESC-RGCs—mouse embryonic stem cell derived RGCs; rRGCs—purified primary rat RGCs; ONC—optic nerve crush; ONH—optic nerve head; NHP—nonhuman primate; WGA—wheat germ agglutinin.

## Data Availability

Not applicable.
